# Utilizing a Video-Based Learning Platform for Teaching Breastfeeding Medicine

**DOI:** 10.7759/cureus.31327

**Published:** 2022-11-10

**Authors:** J.D. Hammond, James Brucker, Laura Seul, Mark Adler, Alanna Higgins Joyce

**Affiliations:** 1 Pediatrics, Northwestern University Feinberg School of Medicine, Chicago, USA; 2 Medical Education, Northwestern University Feinberg School of Medicine, Chicago, USA

**Keywords:** pediatric nutrition, woman’s health, newborn and child health, newborn, breastfeeding education, breastfeeding medicine, lactation consultant, lactation

## Abstract

The American Academy of Pediatrics (AAP) supports exclusive breastfeeding of infants. However, conversations surrounding breastfeeding can be sensitive in nature and cause discomfort for both learners and parents. Additionally, bedside teaching of breastfeeding medicine is a relatively large time commitment which can be difficult for learners rotating through busy delivery centers. These factors along with others have led to known knowledge gaps in medical students, residents, fellows, and even attending knowledge of skill-based breastfeeding competencies supported by the AAP. We aimed to address these gaps by creating a video-based breastfeeding education module working in collaboration with certified lactation consultants at the largest birthing center in Illinois, United States. This technical report describes the utilization of Panopto audio-visual software (Panopto Inc., Seattle, Washington, United States) to successfully create a video-based curriculum for teaching breastfeeding medicine.

## Introduction

The American Academy of Pediatrics (AAP) Policy Statement on Breastfeeding officially recommends “exclusive breastfeeding for 6 months” and “as long as mutually desired by mother and child for two years and beyond" [1]. They advise that physicians working with infants or new parents should be “knowledgeable in the principles and management of lactation and breastfeeding” and “develop skills necessary for assessing the adequacy of breastfeeding” [1]. Despite this policy statement, studies have shown that physicians lack confidence in addressing breastfeeding topics with new parents. Aligned with these statistics, multiple studies have shown that physicians’ overall knowledge of breastfeeding medicine is inadequate and that improvements may lead to breastfeeding success [2,3,4]. 

Developing a strong foundation for breastfeeding principles begins in medical school and continues through residency and fellowship learning. Early opportunities to educate students may lead to better doctor-patient interactions around lactation, with the potential for higher rates of breastfeeding success. However, curriculum reviews performed at United States (US) and Canadian residency programs, as well as medical schools, demonstrate large gaps in current curricula [5,6,7].^ ^ In one study, students demonstrated suboptimal knowledge of the basic principles of breastfeeding medicine at the completion of their pediatric clerkship [8]. Additional studies have identified poor competency in obstetrician-gynecology residents, pediatricians, and family practitioners alike at explaining common breastfeeding problems encountered by patients [9,10].^ ^This technical report outlines the use of a popular audio-visual software system to create learning modules to address these gaps. It is appropriate for medical student learners, pediatric and obstetrician-gynecology residents, family medicine residents, and any practitioners interested in working with newborns or their parents.

## Technical report

Methods

This video-based curriculum was developed by a senior pediatric resident with a demonstrated interest in newborn medicine, along with the support of certified lactation consultants and pediatric faculty interested in novel adult learning methodology. The video lectures included audio material presented by the senior resident as well as embedded videos demonstrating multiple skill-based competencies required to successfully assist and troubleshoot mothers with breastfeeding at the bedside. The curriculum was provided to medical students at the largest birthing center in Illinois, US, for use prior to their newborn medicine rotations. 

Setting and equipment

**Figure 1:**
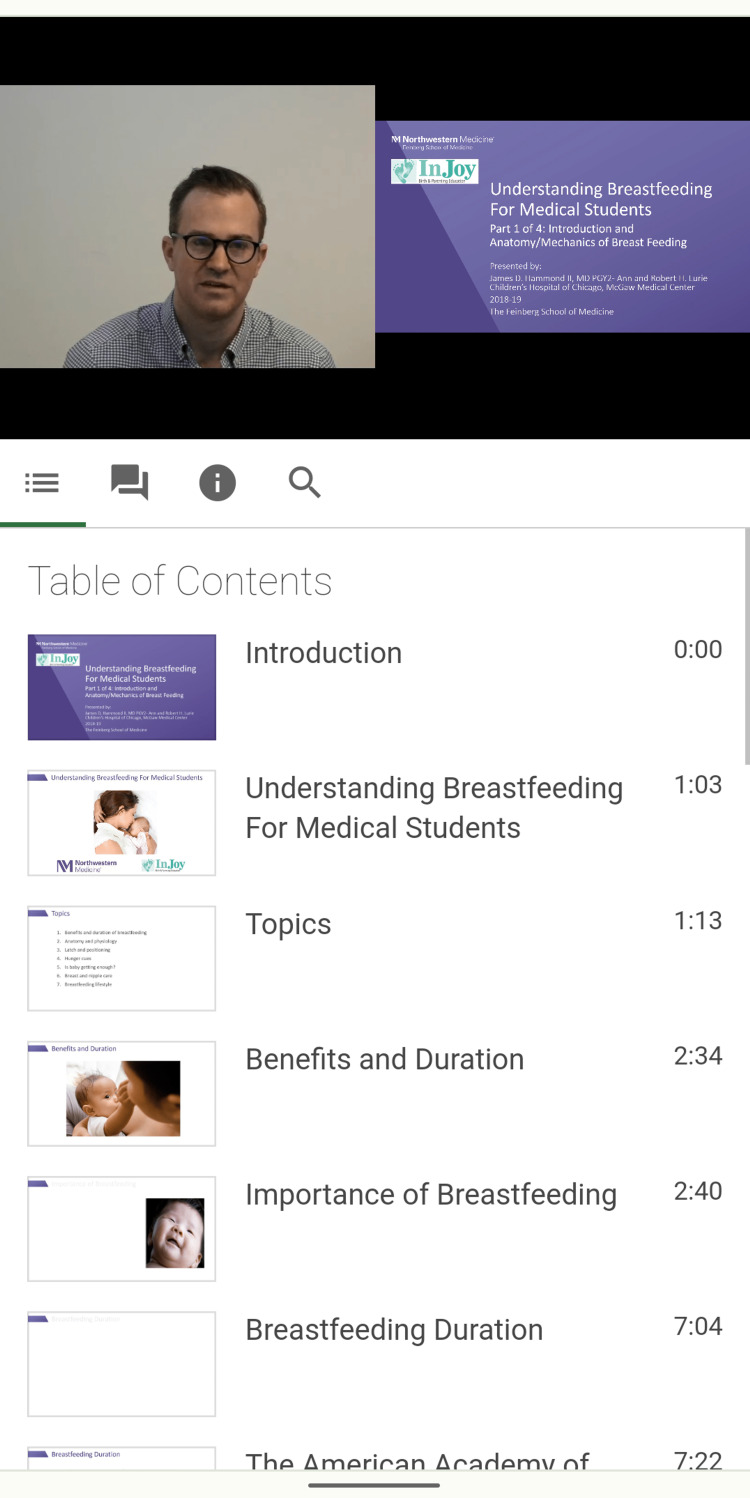
Example of the Panopto Video Learning User Interface (Panopto Inc., Seattle, Washington, United States)

The lectures were created in the audio-visual department of the medical school and recorded using Microsoft PowerPoint and the Panopto Video Learning software platform (Panopto Inc., Seattle, Washington, US; Figure 1). Support for the creation of the videos was provided by the medical school audio-visual department experts. The videos were then uploaded to a password-protected website for dissemination to interested parties. This allowed the video lectures to be accessible 24/7 from the comfort of home. 

Participants

We provided this curriculum to medical students in both an in-person lecture series as well as access to the videos online. Participants were given access to video lectures prior to starting their newborn medicine rotation and had access for the duration of their pediatric clerkship. 

Video summaries

**Figure 2:**
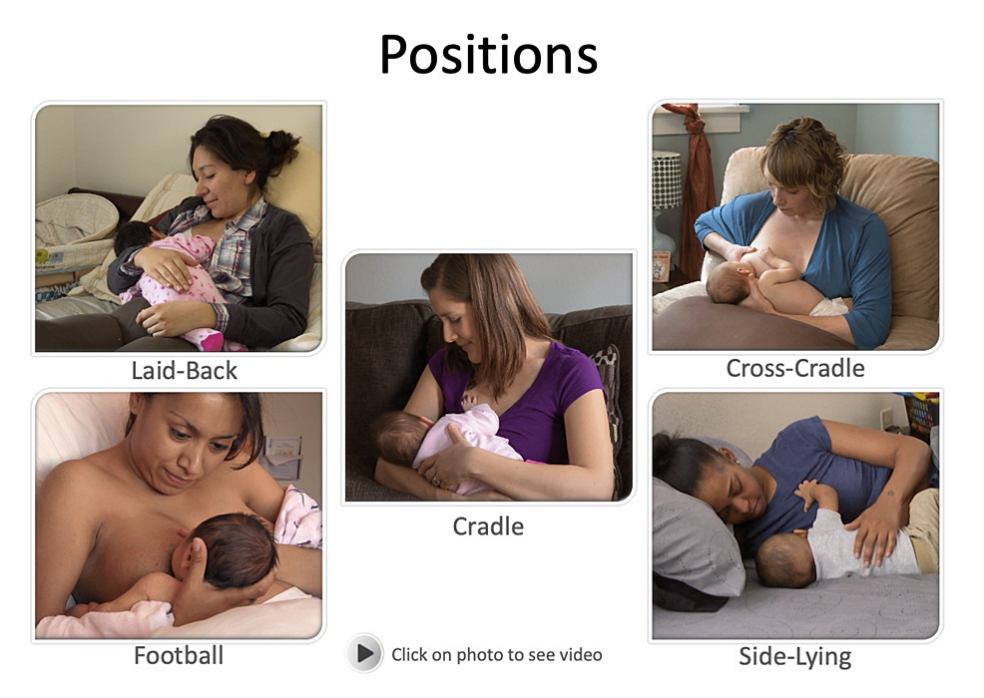
Videos demonstrating proper breastfeeding positioning

**Figure 3:**
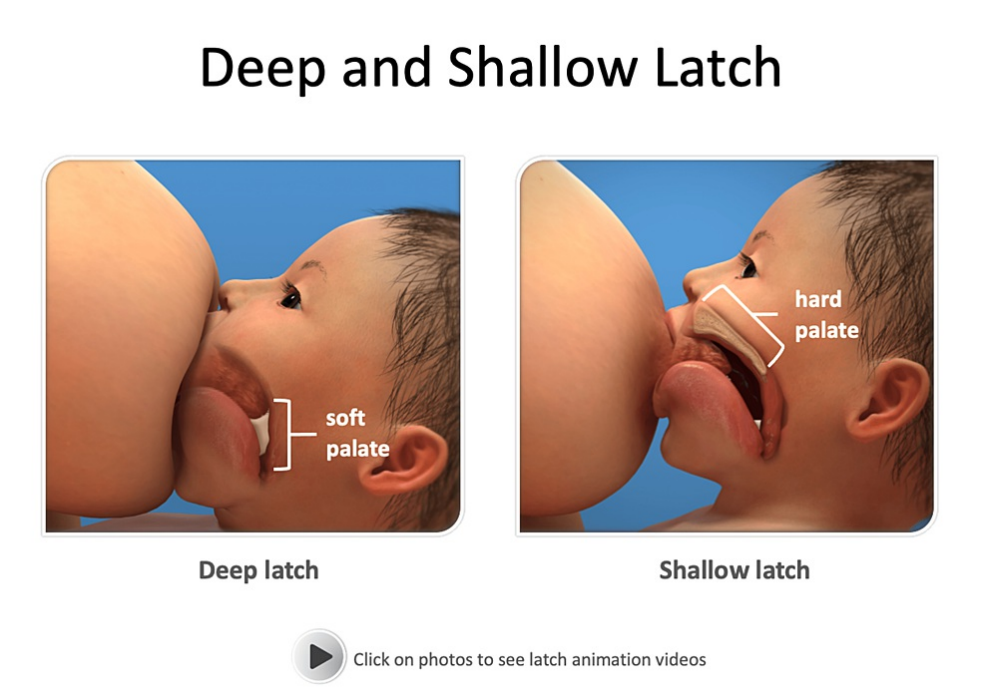
Videos demonstrating successful infant latch

**Figure 4:**
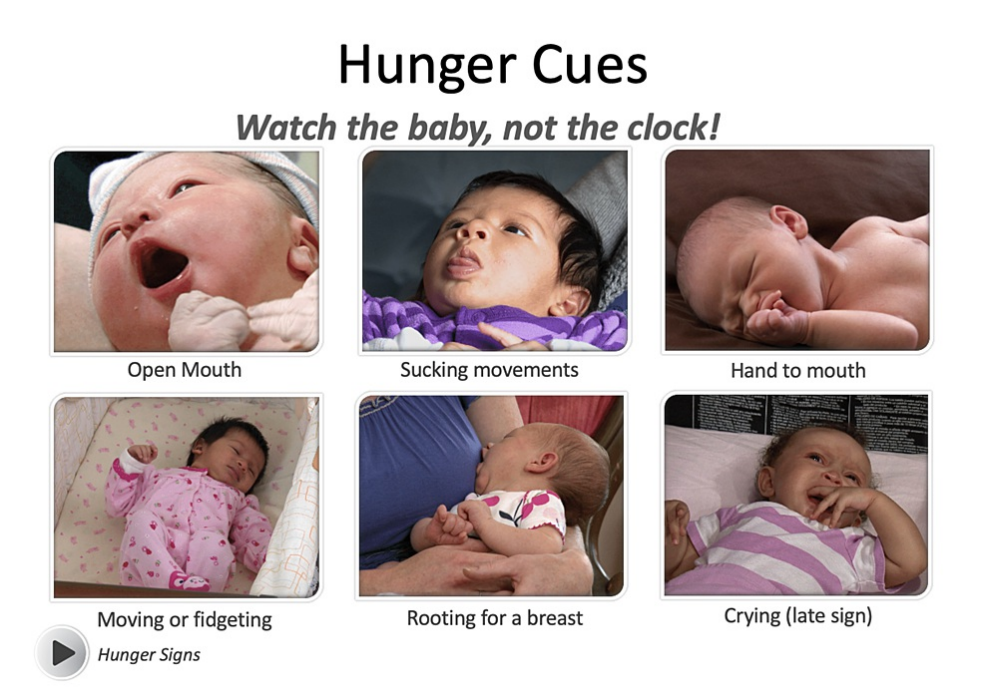
Videos demonstrating infant hunger cues

A total of four separate video lectures were recorded with Panopto Video Learning software targeting the 12 knowledge and skill-based competencies for providers previously identified by the US Breastfeeding Committee/World Health Organization [9]. Topics addressed included the benefits of breastfeeding, anatomy and physiology, proper latch and positioning, identifying hunger cues and adequate intake, and how to provide care for common problems that arise for breastfeeding mothers. Embedded videos were utilized to demonstrate commonly utilized positions for breastfeeding (Figure 2), effective latch characteristics (Figure 3), and identifying hunger cues (Figure 4). 

Assessment 

Participants were given a voluntary survey consisting of six Multiple Choice Questions, six Anchored-Rating Scale Measures (Confidence Levels 1=Least through 5=Most), and five Self-Reported Experiential Measures (Figures in Appendices). Multiple choice questions were adapted from similar questions administered by the AAP to residents and fellows designed to test their overall knowledge of common breastfeeding principles. The anchored-rating scale and experiential measures were developed by our team to assess how confident participants were with individual skills and how often they were given a chance to use those skills during their rotation. 

## Discussion

Limited materials exist on subscription-based platforms and are currently lacking in their inclusion of clinically relevant, skill-based videos [11]. The AAP recently updated its policy on breastfeeding medicine to encourage the continuation of breastfeeding through two years of age as desired by mother and infant [12]. This change places even more responsibility on pediatricians and family practitioners for supporting breastfeeding mothers and ensuring successful breastfeeding practices. Unfortunately, learners across multiple specialties are lacking exposure to counseling breastfeeding parents through the course of their medical school and residency rotations and report low confidence in their ability to counsel on the more practical aspects of breastfeeding medicine. These findings may be related to several factors that are not limited to our institution. Concerns in the era of COVID-19 along with evolving restrictions on student autonomy in healthcare institutions may interfere with hands-on lactation learning. Other variables such as the volume of newborns in each center, and the individual emphasis residents and attendings might differentially place on teaching breastfeeding topics to students on a given rotation also may decrease exposure to hands-on learning in the course of a clerkship. We feel students may benefit from more hands-on learning whether that comes from clinical exposure versus mixed-media learning using video lectures. Our mixed-media virtual lecture series can provide a convenient, easily accessible, remote option to simulate those hands-on experiences, improve student learning, and promote breastfeeding at our institution and across the country.

While this module was implemented with medical students, several residents, fellows, and attending-level providers expressed interest in the module. Since the curriculum can easily be stored on a centralized platform and easily disseminated to interested parties, it can be implemented in a variety of settings for a diverse group of learners. We can do a better job at educating learners on not only the risks and benefits of breastfeeding but the skill-based components as well, and our videos will help address that goal. Given that successful breastfeeding is a public health and economic issue, it is imperative that the medical community places a larger emphasis on educating future physicians and caregivers on breastfeeding medicine. 

## Conclusions

Panopto Video Learning software can be used to create mixed media lectures that can be distributed to students for remote and home learning. The combination of embedded videos and recorded audio is well received by adult learners of all levels and provides an alternative to bedside teaching when such opportunities are limited. 

## Figures and Tables

**Figure 5:**
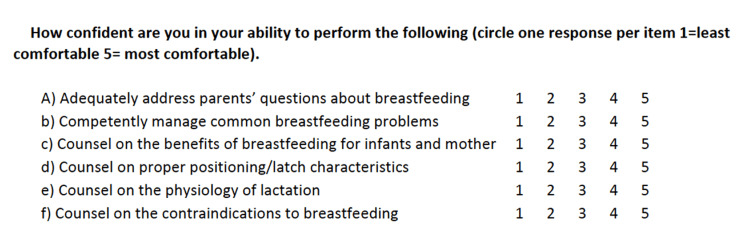
Student comfort with breastfeeding principles

**Figure 6:**
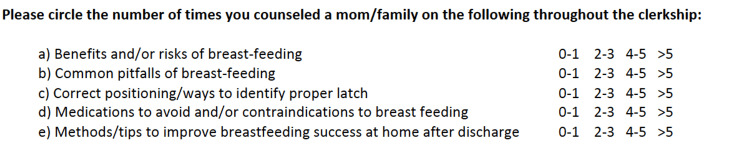
Student experience with breastfeeding topics at the bedside
